# TheLNet270v1 – A Novel Deep-Network Architecture for the Automatic Classification of Thermal Images for Greenhouse Plants

**DOI:** 10.3389/fpls.2021.630425

**Published:** 2021-07-01

**Authors:** Md. Parvez Islam, Yuka Nakano, Unseok Lee, Keinichi Tokuda, Nobuo Kochi

**Affiliations:** ^1^Agricultural AI Research Promotion Office, RCAIT, National Agriculture and Food Research Organization (NARO), Tsukuba, Japan; ^2^Institute of Vegetable and Flower Research, NARO, Tsukuba, Japan

**Keywords:** deep learning, network architecture, classification, segmentation, thermal image

## Abstract

The real challenge for separating leaf pixels from background pixels in thermal images is associated with various factors such as the amount of emitted and reflected thermal radiation from the targeted plant, absorption of reflected radiation by the humidity of the greenhouse, and the outside environment. We proposed TheLNet270v1 (thermal leaf network with 270 layers version 1) to recover the leaf canopy from its background in real time with higher accuracy than previous systems. The proposed network had an accuracy of 91% (mean boundary F1 score or BF score) to distinguish canopy pixels from background pixels and then segment the image into two classes: leaf and background. We evaluated the classification (segment) performance by using more than 13,766 images and obtained 95.75% training and 95.23% validation accuracies without overfitting issues. This research aimed to develop a deep learning technique for the automatic segmentation of thermal images to continuously monitor the canopy surface temperature inside a greenhouse.

## Introduction

**Figure 1:**
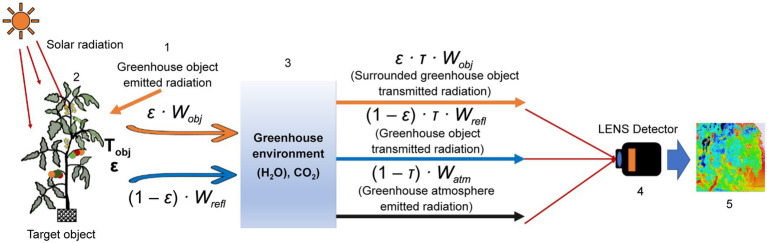
A schematic representation of a thermal camera working principle. 1: surroundings, 2: object, 3: atmosphere, 4: thermal camera, and 5: thermal image.

Leaf surface and internal structure changes are due to adverse growth, stomatal resistance, diseases, leaf angles, depth of the canopy, and water stress conditions, which alter the absorbance-reflection process of solar radiation (Lili et al., 1991; Kraft et al., 1996; Raza et al., 2015). Thermography detected this reflected (emitted) long-wave infrared (8–14 μm), then converted it into thermal images, and a false-color gradient demonstrated the temperature level of the plant leaves of canopies (Chaerle and Van Der Straeten, 2000). Figure 1 shows the working principle of a thermal camera.

**Figure 2:**
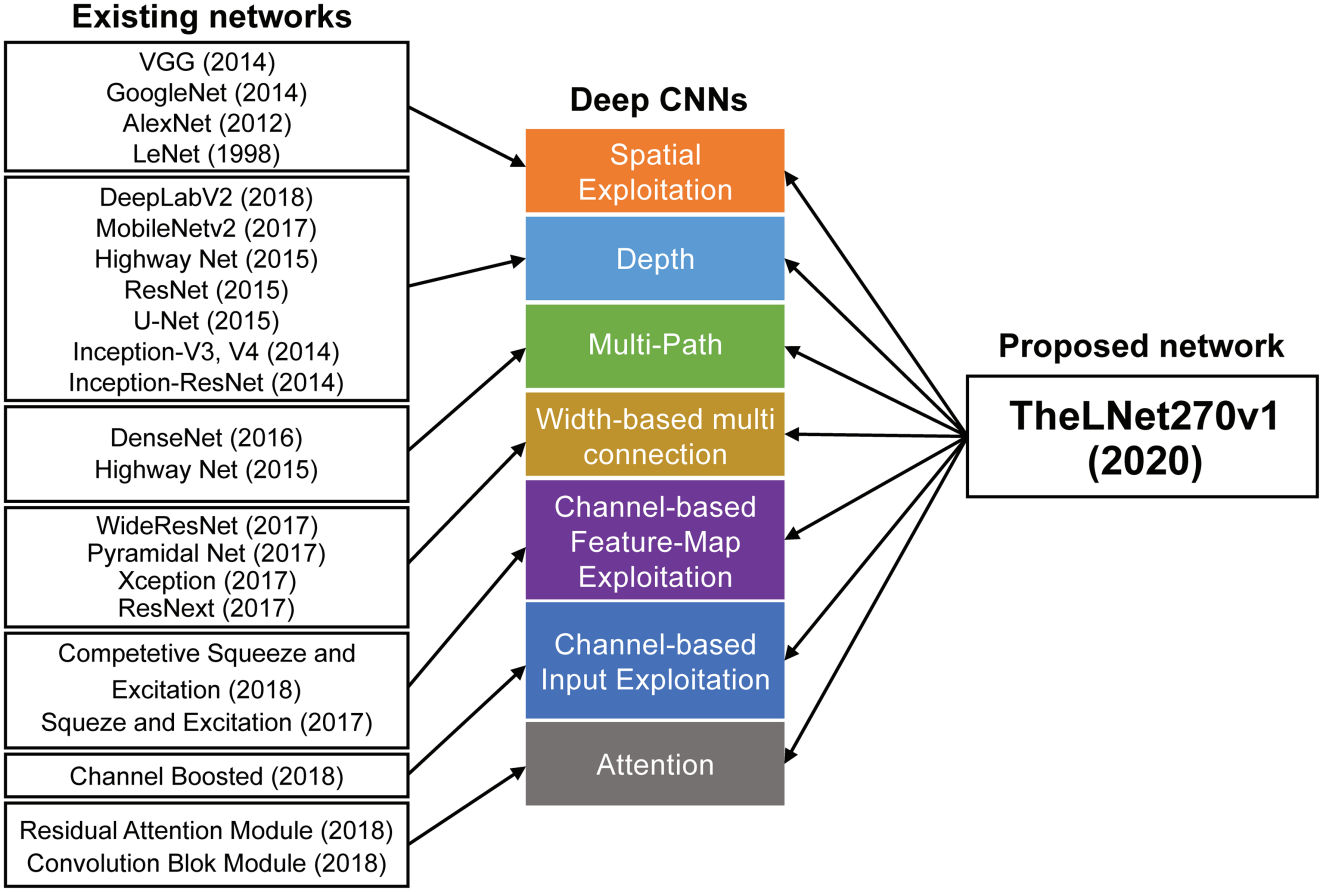
Classification of available and proposed deep network architecture.

Over the last few years, the advancement of fast computing power, low-cost imaging systems with image processing software, and deep learning (DL) techniques have allowed for nondestructive disease diagnosis and detection of various stress conditions of plants in a timely manner (Liu and Wang, 2020). The DL based on a convolution neural network (CNN) is the successor of traditional machine learning approaches that can learn features with greater precision and accuracy by activating maximum networkability (Christopher et al., 2018). Bengio (2009) compared CNN-based DL with the Neocortex of the human brain, which learns response-based features dynamically from images. CNN-based DL acquires hierarchical features and emphasizes nonlinear filters of the depth of the deep network structure for learning, and after that solves problem-specific tasks such as image classification, semantic segmentation (pixel-based classification), object detection, video processing, speech recognition, and natural language processing (Simonyan and Zisserman, 2014; Singh et al., 2018; Liu and Wang, 2020). Khan et al. (2020) classified deep network architectures into seven classes: spatial exploitation, depth, multi-path, width, feature-map exploitation, channel boosting, and attention-based CNNs. Figure 2 demonstrates the classification of various deep network architectures along with the proposed TheLNet270v1. Shin et al. (2016) stated that a filter termed as a channel in a CNN can extract different levels of information (from fine-grained to coarse-grained) based on their sizes (small to large sizes). Simonyan and Zisserman (2015) and Khan et al. (2020) stated that the deep DL architecture has an advantage over the shallow depth DL architecture, which can learn complex representations at different levels of abstraction and thus increase the classification accuracy. According to Szegedy et al. (2015), branching within layers can abstract features with various spatial scales. Srivastava et al. (2015), Dong et al. (2016), Larsson et al. (2016), Mao et al. (2016), Dauphin et al. (2017), Huang et al. (2017), Tong et al. (2017), and Kuen et al. (2018) proposed multi-paths or shortcut connections that connect one layer with another layer by skipping some intermediate layers. This allows overpassing some information to another layer and reduces the vanishing gradient problem, which causes a higher training error.

Li et al. (2019) proposed an edge-conditioned convolution neural network for thermal image segmentation with SODA (segment objects in day and night) benchmark for evaluating the thermal image segmentation performance. They used manually annotated synthetically generated thermal images for training the network, which achieved 61.9% mean intersection over union (IoU), a lightly better than network trained with DeepLabv3 algorithm. Choi et al. (2016) developed a CNN-based thermal Image Enhancement technique for improving low-resolution thermal camera recognition tasks. The lightweight structure of the shallow convolutional neural network requires less CPU memory. In their architecture, they cropped a low-resolution thermal image with a uniform stride and used a bi-cubic interpolation method to upscale it. Chen et al. (2019) revealed a Fletcher-Reeves algorithm-based CNN model for hyperspectral image classification with 80.7% accuracy which outperforms other traditional CNN due to the advances in batch computing adaptability and convergence speed. Grbovic et al. (2019) reported a thermal-RGB image-based wheat-ears detection system for automatic counting wheat wars under outdoor conditions. They applied blocks of convolutional layers, each with an activated function for counting the wheat ears, which achieved 75.63 and 68.46% F1 Score for segmenting thermal and RGB images. Furthermore, achieved 89.22% accuracy for counting the wheat ears. Another study reported by Zhang et al. (2019) used a group of neurons termed as a capsule or vector for replacing traditional neurons and achieved equivariance by successfully encoding spatial information and properties of an input image. Bhattarai and Martínez-Ramón (2020) identified and classified objects in real time from thermal cameras carried by firefighters. The detection accuracy reported by authors varied from 70 to 95%, which depends on the depth of the convolution network layer.

Fuentes-Pacheco et al. (2019) developed a CNN with an encoder–decoder function which used top-view RGB images of fig plants and achieved a mean 93.85% segmentation accuracy under variable visual fig leaves the appearance and complex background. There are various DL architectures, such as LeNet, AlexNet, VGG, GoogleNet, YOLOv, Inception, and SqueezeNet, which are widely used for image classification and object detection. However, ResNet, U-Net, DeepLabv3, and MobileNet are mostly used for semantic segmentation (pixel) – based image (RGB) classification (Boulent et al., 2019; Saleem et al., 2019). In agriculture, the high or low thermal dynamic changes during sunny–cloudy–rainy days and nights make it difficult to spatially process bulk thermal images, such as separation of leaf/canopy pixels from background pixels (Cho et al., 2017; Salgadoe et al., 2019). To solve this classification challenge, the author proposed a new DL architecture with several components [convolutions, grouped convolution, transposed convolution, batch normalization, rectified linear unit (ReLU), max pooling, depth concatenation, element-wise addition, 2D crop, softmax, and classification output layer]. The aim of this study was to develop a DL architecture and demonstrate the learning ability of the DL architecture to separate the leaf/leaf canopy from a greenhouse background (ground, windows, roof, etc.) in thermal images under various environmental conditions (sunny, cloudy, and rainy: day or/and night).

## Materials and Methods

### Thermal Image Acquisition System

**Figure 3:**
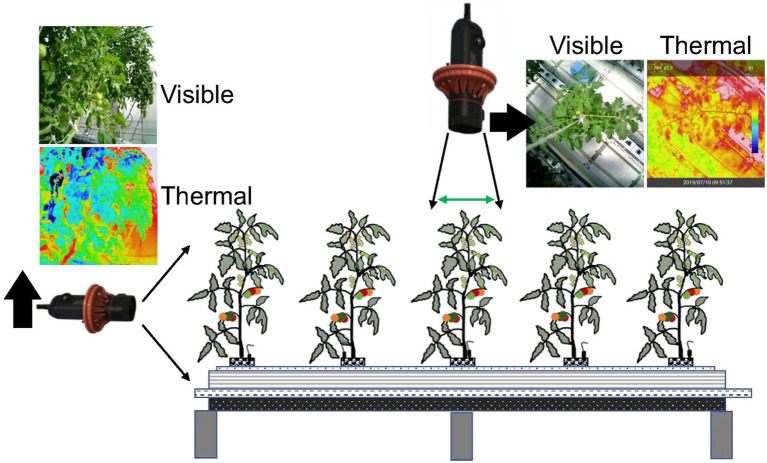
Thermal image acquisition technique.

The study was conducted in the greenhouse of the Vegetable and Flower Research Division, National Agriculture and Food Research Organization (NARO) in Tsukuba, Ibaraki, Japan. The Japanese cultivar “CF Momotaro York” (Takii Seeds Co., Ltd., Kyoto, Japan) of tomato (*Solanum lycopersicum*) grown in a Rockwool system was used for this experiment. The image data collection period ran from October 16, 2019 to September 30, 2020. The air temperature and relative humidity at 1.2 m above the ground surface ranged between 8.6 and 37.5°C, 32 and 96% from October 16, 2019 to April 16, 2020. The air temperature and relative humidity at 1.2 m above the ground surface ranged between 9.6 and 39.3°C, 34 and 95% from August 7, 2020 to October 28, 2020. Thermal images with 1040 × 780 pixel resolution (screen) were obtained, as shown in Figure 3, using a compact long-wave thermal camera [Thermo FLEX F50B-ONL (Nippon Avionics Co., Ltd., Yokohama, Japan)] under various environmental conditions at a minimum distance of 0.3 m from the top and maximum 2 m from the side of the targeted tomato plant.

All images were stored in a 24-bit thermal image format. The emissivity range of the thermal camera is 0.1 to 1. In this experiment, the emissivity of the tomato leaf was considered to be 0.98 (López et al., 2012). The technical specifications of the thermal camera are listed in Table 1.

**Table 1:** Technical specification of the thermal camera (Thermo FLEX F50B-ONL).

Field of view, °	Focus	Spectral range, μm	Frame rate, Hz	Sensitivity, °C	Accuracy
70°×70°	Focus free	8~14	7.5	0.05°C at 30°C	±2°C or ±2% for 0–40°C (other conditions: ±4°C or ±4%)

### Image Dataset Preparation

**Figure 4:**
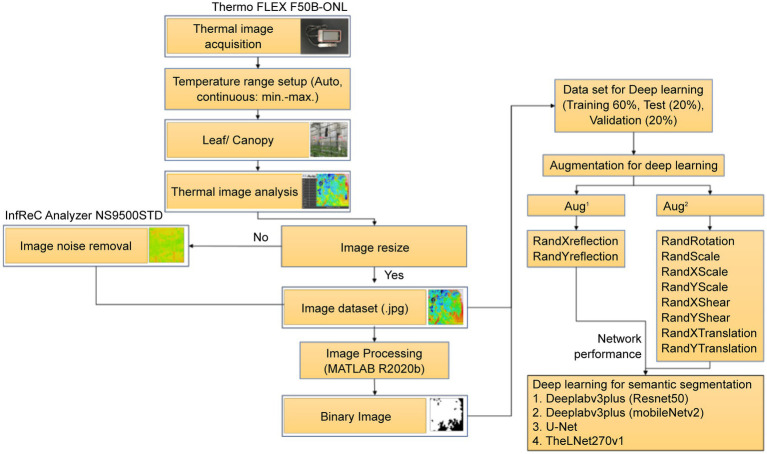
Schematic diagram of the image dataset preparation.

Figure 4 demonstrates the schematic diagram of the image dataset preparation for network analysis.

**Figure 5:**
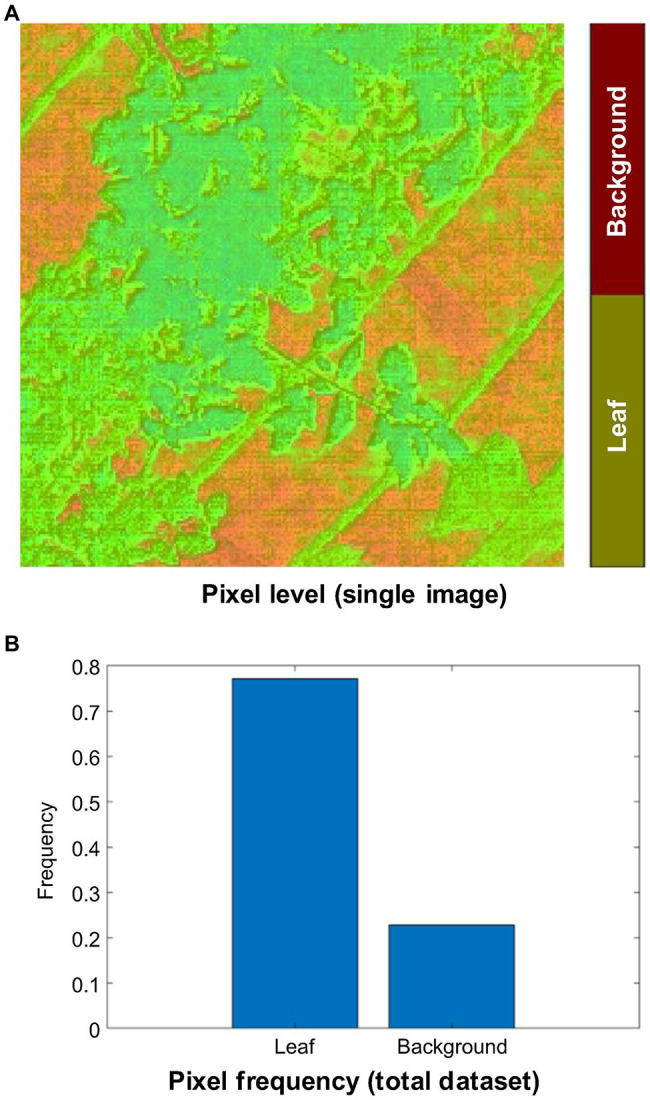
Pixel classification of the thermal image dataset. **(A)** Manual annotation. **(B)** Frequency level of the annotated leaf and background pixels within total thermal image dataset.

In total, 13,766 thermal images were obtained during this experiment. The thermal images were resized into their original spatial resolution (240 × 240 pixels), and denoising (manipulation of scale and emissivity) was performed by a thermal imaging processing software (InfReC Analyzer NS9500STD for F50, Nippon Avionics Co., Ltd.) to meet the network input dimension (240 × 240 pixels) requirements. Furthermore, Image Segmenter (Image Processing and Computer Vision Toolbox, MATLAB R2020a) was used to convert the pixels of each thermal image into two groups manually: leaf (255) and background (0) as shown in Figure 5A. These pixel values were stored in binary images. The frequency levels of the leaf and background pixels within the total thermal image datasets were 77 and 23%, respectively (Figure 5B). In this experiment, 60% of the randomly selected images (thermal images and binary images) were used for training, 20% for validation, and 20% for test purposes.

**Figure 6:**
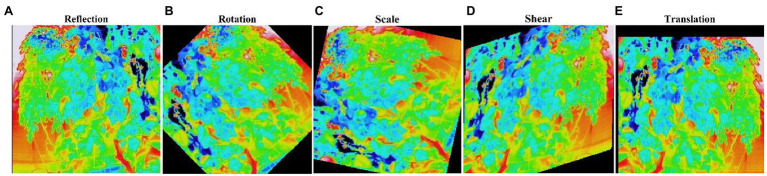
Augmented image dataset. The statistics of the image datasets used for the deep learning (DL) network analysis are shown in Table 2. (A) Reflection; (B) Rotation; (C) Scale; (D) Shear; and (E) Translation.

The image dataset was augmented to increase the amount and type of variation within the training image data to prevent overfitting and generalizing the model performance (Figures 6A–E). Table 2 shows the number of image datasets used for deep learning analysis. First, we augmented the image data, including random reflection in the X and Y directions [(aug^1^)]. This dataset was used for the network performance study. Furthermore, for comparative analysis, we also augmented the thermal image dataset with the other four options (aug^2^), as shown in Table 3.

**Table 2:** The number of image datasets used for the DL analysis.

Condition	Original dataset	Binary dataset	Training	Validation	Test
Total image number	13,766	13,766	8,260	2,753	2,753

**Table 3:** Properties of the augmented datasets for DL analysis.

Augmentation options		Training option	Visualization
RandXreflection	1	Aug^1^	Figure 6A
RandYreflection	1		
RandRotation	[−90 90]	Aug^2^	Figure 6B
RandScale	[1 1]		
RandXScale	[0.8 1.2]		Figure 6C
RandYScale	[0.8 1.2]		
RandXShear	[−20 20]		Figure 6D
RandYShear	[−20 20]		
RandXTranslation	[−10 20]		Figure 6E
RandYTranslation	[−10 20]		

### Network Architecture

**Figure 7:**
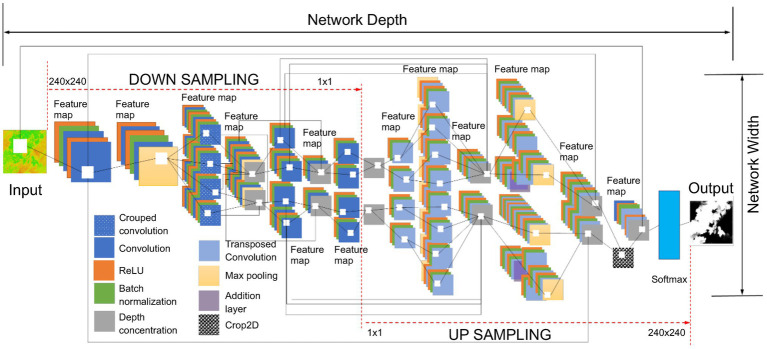
The basic network architecture of the TheLNet270v1.

Figure 7 demonstrates the basic network architecture of the TheLNet270v1, which is a combination of the semantic segmentation-based network (convolution layers) and classification-based network (softmax). The convolution layer of the proposed network extracts the higher-level features from input images with multiple smaller filter sizes (3 × 3 × 3 × 32). The smaller filter size of the convolution layer has a strong generalization ability when the same types of objects within an image are conglutinated with each other (Zhang et al., 2020). This capability effectively improves network learning performance. According to Nair and Hinton (2010), the ReLUs activation function added non-linearities to the model, converted values less than zero to zero for each element of the input, transformed the summed weighted input from the node into output, and allowed models to learn faster with higher accuracy. The batch normalization layer increases the network stability and normalizes the output of a previous activation layer by subtracting the batch mean and dividing by the batch SD (Ioffe and Szegedy, 2015). Krizhevsky et al. (2012) introduced grouped convolution for training AlexNet with less powerful GPUs with limited RAM. It is also termed as convolutions in parallel as this layer separates input channels into groups by applying sliding convolution filters (vertically and horizontally), computing the input and weights, adding a bias, and finally combining the convolutions for each group independently (Xavier and Bengio, 2010; He et al., 2016a). We included grouped convolution to increase the width of the network without hampering computational power. According to Scherer et al. (2010) and Zhang et al. (2020), the max-pooling layer simplifies the network complexity by compressing and extracting the main features, ensuring feature position and rotation invariance, and rotation reduced computing time. A 2D image cropping layer crops images at the center to explore contextual features (Blaschke, 2010). The last convolution layer has two outputs corresponding to two classes with a ReLU activation followed by a batch normalization layer with 16 filters. The output of the last convolution layer is fed into the softmax layer for calculating the probability of the output classification layer. Finally, these expanded features are passed to the classification layer for classification (Krizhevsky et al., 2012). Therefore, the depth of the DL architecture is fixed to 270 layers and accurately optimized based on training performance. The characteristics of the TheLNet270v1 architecture are shown in Table 4.

**Table 4:** Characteristics of the TheLNet270v1 architecture.

Layers name	Total number of layers
Image input	1
Convolution	31
ReLU	83
Batch normalization	71
Max pooling	15
Transposed convolution	49
Addition layer	2
Grouped convolution	4
Depth concatenation	11
Crop2D	1
Softmax	1
Pixel-classification (output)	1

### Network Parameters

The TheLNet270v1 was trained on a FUJITSU SHIHO Supercomputer equipped with TESLA V100-SXM2 32GB and CUDA version 10.2, DL, and parallel computing toolbox (MATLAB R2020a). The adaptive moment estimation (ADAM) algorithm was used to optimize the network weights. The transfer learning parameters applied for training the TheLNet270v1 were as follows: training option: Adam; validation frequency: 10; mini-batch size: 50/70/90/128/156/220/240/260/290/320; max epoch: 5/12/20/30/40; learn rate schedule: piecewise; shuffle: every-epoch; initial learn rate: 0.001; epsilon: 1e-08. ADAM was used to optimize the network weights. Table 5 shows the hyperparameter optimization parameter for the TheLNet270v1 training.

**Table 5:** Hyperparameter optimization parameter.

Training options: adam	Execution environment: parallel
Learn rate schedule: piecewise	Validation patience: Inf
Shuffle: every-epoch	Epsilon:1e-8
Verbose: false	Initial learn rate: 1.0000e-03
Validation frequency:10	Learn rate drop factor: 0.1000
Gradient decay factor: 0.9000	Learn rate drop period: 10
Squared gradient decay factor: 0.9990	Gradient threshold method: l2norm
L2 regularization: 1.0000e-04	Verbose frequency: 50
Gradient threshold: Inf	Dispatch in background: 0
Sequence padding value: 0	Reset input normalization: 1
Sequence length: longest	Sequence padding direction: right

### Comparative Analysis and Evaluation Metrics

Currently, MobileNetv2 is widely used in low-powered mobile devices for image recognition or classification tasks because of its simple network architecture and lower computational complexity (Wong et al., 2020). He et al. (2016a) first introduced ResNet with cross-layer connectivity in a CNN, which sped up the convergence of deep neural networks, solved the vanishing gradient problem by actively deploying special skip connections and a batch normalization layer and 20 and 8 times deeper than AlexNet and VGG. On the other hand, U-Net is mostly used in high-powered fixed devices because of its complex network architecture. It is widely used for biomedical image segmentation and classification purposes (Ronneberger et al., 2015). The bottleneck layer between the contracting and expanding paths of the U-Net architecture increased the network depth and was regularized by dropout to solve the overfitting issue during the network learning process (Krizhevsky et al., 2012; He et al., 2016b). Giusti et al. (2013) stated that Deeplabv3plus employs atrous convolution or dilated convolutions in parallel or in cascade to extract dense features at multiple scales with better-stored information capability. TheLNet270v1 is designed so that it can be used in both low-powered mobile or high-powered fixed devices. There are several performance metrics such as training/validation/test accuracy (shows the percentage of correctly classified pixels), global accuracy (measuring ratio of correctly classified pixels to the total number of pixels), mean accuracy (measuring the percentage of correctly identified pixels for each class), confusion metrics, validation loss, training time, IoU/Jaccard index (measuring the amount of overlap per predicted class), weighted IoU (measuring the average IoU of each class), BF score (Boundary F1 – measuring the quality of the predicted boundary with the ground truth boundary), etc. are used for quantifying TheLNet270v1 accuracy and network efficiency. The same performance metrics were also evaluated on Deeplabv3plus (with a pretrained network MobileNetv2 and ResNet-50) and U-Net for comparative analysis.

## Results and Discussion

Image datasets are augmented into two categories for network training. The augmented dataset^1^ and augmented dataset^2^, as shown in Table 6, are both used for performance study and comparative analysis.

**Table 6:** The image datasets for performance study and comparative analysis.

Image data	Original image	Binary image	Total image	Comments
Original dataset	13,766 × 1	13,766 × 1	27,532	-
Augmented dataset^1^	13,766 × 2	13,766 × 2	55,064	Original condition + XYReflection
Augmented dataset^2^	13,766 × 6	13,766 × 6	82,596	Original condition + XYReflection + Rotation + Scale + XYScale + XYShear + XY Translations

### Feature Extraction and Activation for Visualization

**Figure 8:**
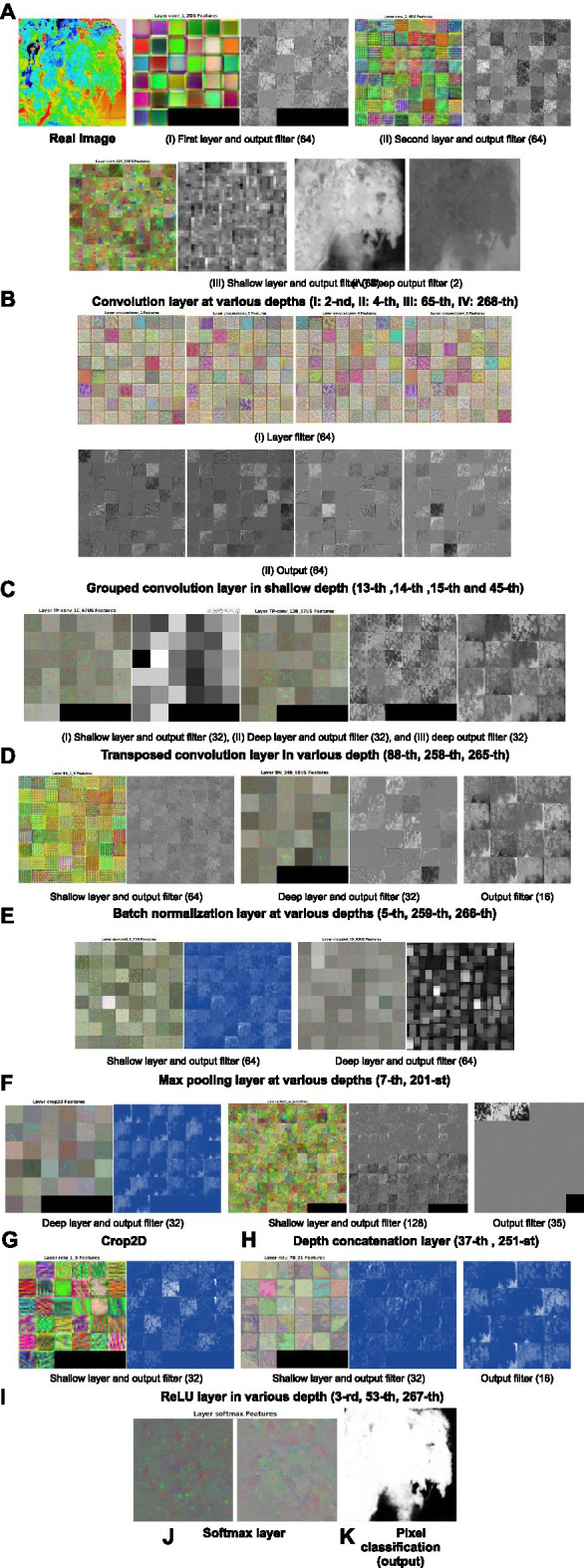
Feature maps and visualization of the network. (A) Visualization of the first and second layer. (B) Convolution layer at various depths. (C) Grouped convolution layer at various depths. (D) Transposed convolution layer at various depths. (E) Batch normalization layer at various depths. (F) Max pooling layer at various depths. (G) Visualization of the crop2D. (H) Visualization of the depth concatenation layer. (I) ReLU layer at various depths. (J) Visualization of the softmax layer. (K) Visualization of the output.

Features extracted and visualized from the different depths of the TheLNet270v1 layers after completing the training are shown in Figure 8. Typical looking filters starting from the first layer in Figure 8B(I) show the colorful smooth pixels of each of the 64 filters, to noisy pixels in Figure 8B(II), and then slightly visible some features in Figure 8B(III). The last convolution layer in Figure 8B(IV) finally represents the visible pixel class. In Figures 8C(I,II), identical features of the grouped convolution layer in shallow depth are shown at different positions of an image. Figures 8D–I reveal different structures of the feature maps within each filter and layer, and visualizations show that the feature map is activated on the foreground tomato leaf image, not the background objects. Finally, softmax (Figure 8J) gives a discrete probability for each class (leaf/leaf canopy and background), which is between 0 and 1, and the result is visualized in the pixel classification output layer (Figure 8K), where 1 (white color) means leaf/leaf canopy and 0 means background (black color).

### Performance Metrics

**Figure 9:**
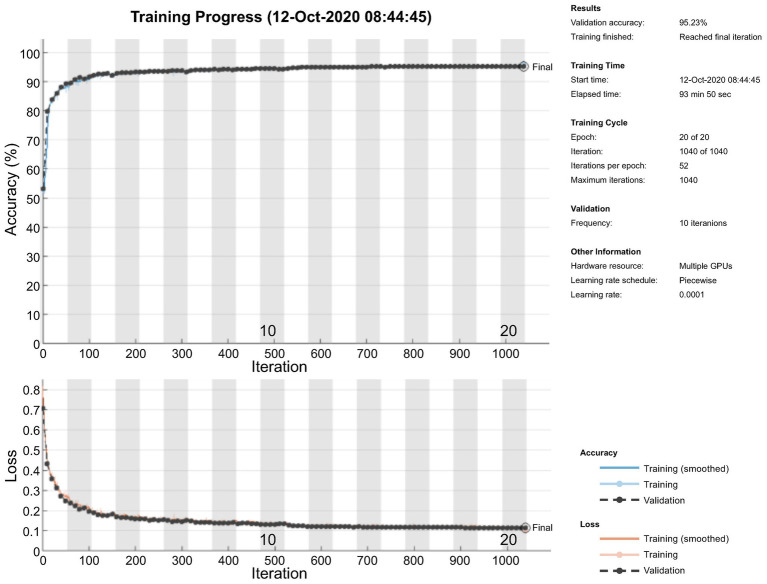
Monitoring of overfitting.

Figure 9 shows the accuracy and loss of the training and validation datasets used to monitor the network overfitting issue. It is clearly visible that the model performs well on both training and validation data sets.

**Figure 10:**
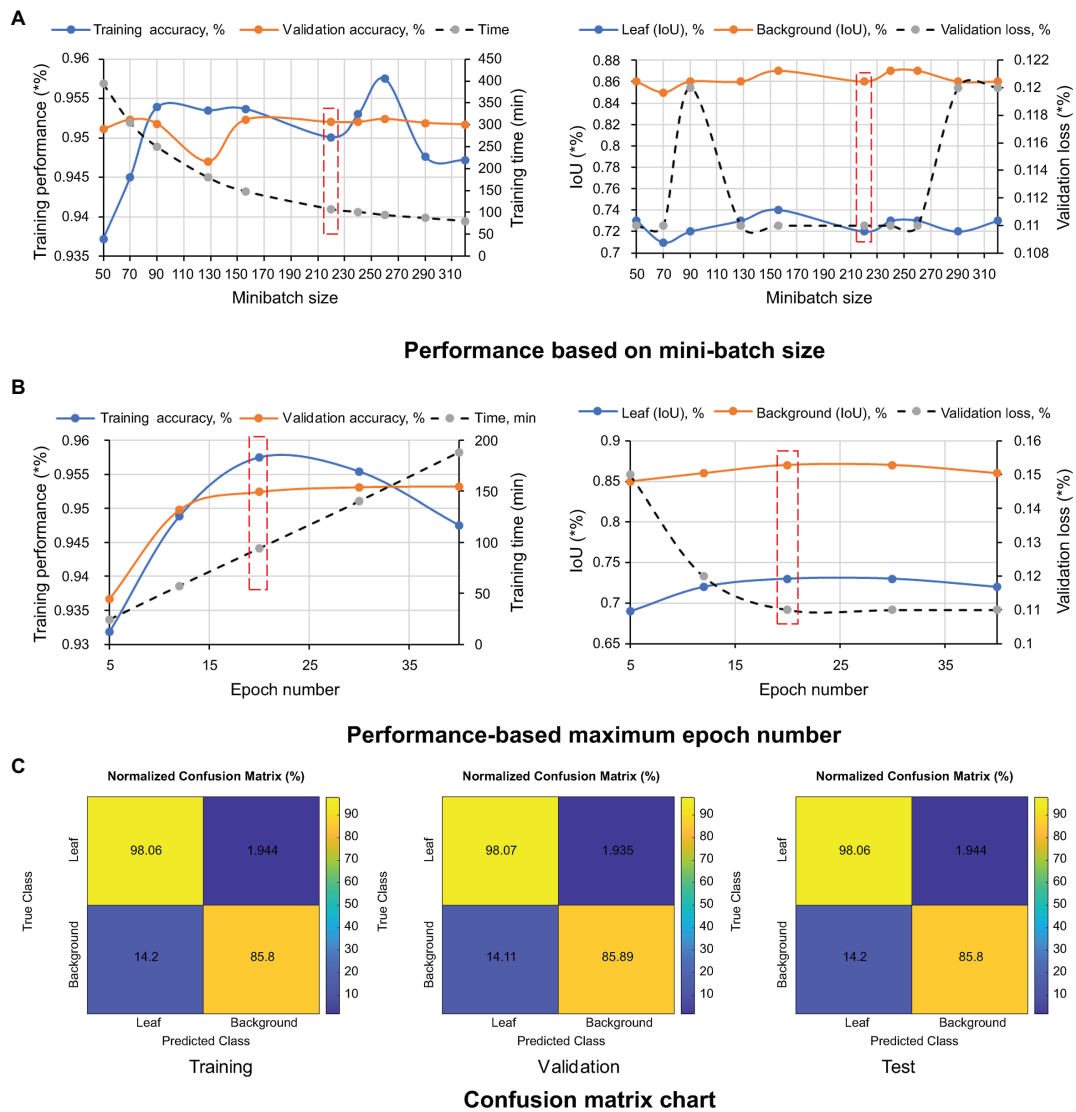
Performance metrics of TheLNet270v1. (A) Performance based on mini-batch size. (B) Performance-based maximum epoch number. (C) Confusion matrix chart.

The pixel-level classification of thermal images by TheLNet270v1 was investigated. A validation accuracy of 95.22% was achieved with a minibatch size of 320, max epoch of 20, and training time of 94.15 min, shown in Figures 10A,B. Under the same conditions, the maximum IoU of 74 and 87% for leaf and background was achieved. During this time, a minimum validation loss of 12% was observed. The confusion matrix is given in terms of percentage and absolute number. It can be seen from the confusion chart in Figure 10C that the higher classification accuracies of 98.07, 98.06, and 98.07% for leaf and 85.89, 85.80, and 85.51% for the background achieved with the training, validation, and testing datasets (Table 6) and demonstrated that the network was well-trained.

Table 7 shows the test results of several other performance metrics such as global accuracy, mean accuracy, weighted IoU, and BF score. A higher value indicates better network performance.

**Table 7:** The performance metrics for image datasets.

Accuracy	Global accuracy, %	Mean accuracy, %	Mean IoU, %	Weighted IoU, %	Mean BFScore, %
Train metrics	94.85	91.99	86.50	90.33	86.42
Validation metrics	94.82	91.69	86.43	90.28	86.34
Test metrics	94.81	91.75	86.20	90.26	86.36

The classification accuracy of each class (leaf and background) is described in Table 8.

**Table 8:** The intersection over union (IoU) and BFScore for each class.

Accuracy	Train metrics			Validation metrics			Test metrics		
	Accuracy	IoU	Mean BFScore	Accuracy	IoU	Mean BFScore	Accuracy	IoU	Mean BFScore
Leaf	97.27	93.57	90.99	97.24	93.54	91.03	97.29	93.56	91.10
Background	86.72	79.42	81.78	86.70	79.33	81.60	86.21	78.84	81.56

**Figure 11:**
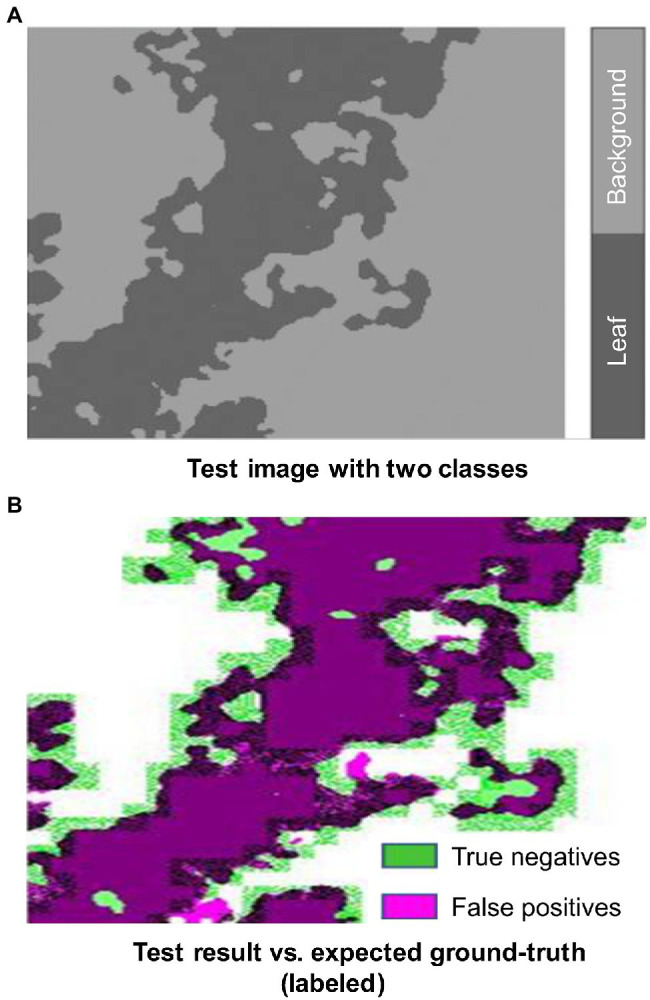
Test image vs. expected ground truth. (A) Test image with two classes. (B) Test results vs. expected ground-truth (labeled).

Figure 11A shows an example of a test image successfully segmented into two classes, in which the dark color area represents leaf and light color background. Figure 11B shows a tiny presence of false positives (magenta color). However, the boundary between leaf and background is marked as green color (true negatives), which described that further refinement is possible if we retrain the network with more image data or images with higher resolutions.

### Comparative Metrics

It is evident from Table 9 that the TheLNet270v1 has a maximum depth layer of 270 with a lower total number of network parameters of 2e + 11, which is lower than Deeplabv3plus (ResNet50) and Deeplabv3plus (MobileNetv2). However, U-Net has a minimum of 46 layers with a higher total number of network parameters of 6e + 06 than TheLNet270v1. However, the training time for all networks (20 epoch, 220 minibatch sizes, and augmented dataset^1^) slightly differed.

**Table 9:** Comparative statistics of the various network architectures.

Network name	Total layers	Total neurons	Total weights	Total biases	Total parameters	Training time, min
Deeplabv3plus (ResNet50)	206	4.00E + 07	6.00E + 06	4.00E + 04	4.00E + 12	80.51
Deeplabv3plus (MobileNetv2)	186	4.00E + 07	7.00E + 06	4.00E + 04	4.00E + 12	94.29
U-Net	46	8.00E + 07	7.00E + 06	3.00E + 03	6.00E + 06	94.16
TheLNet270v1	270	5.97E + 07	1.63E + 06	3.00E + 03	2.00E + 11	94.15

**Figure 12:**
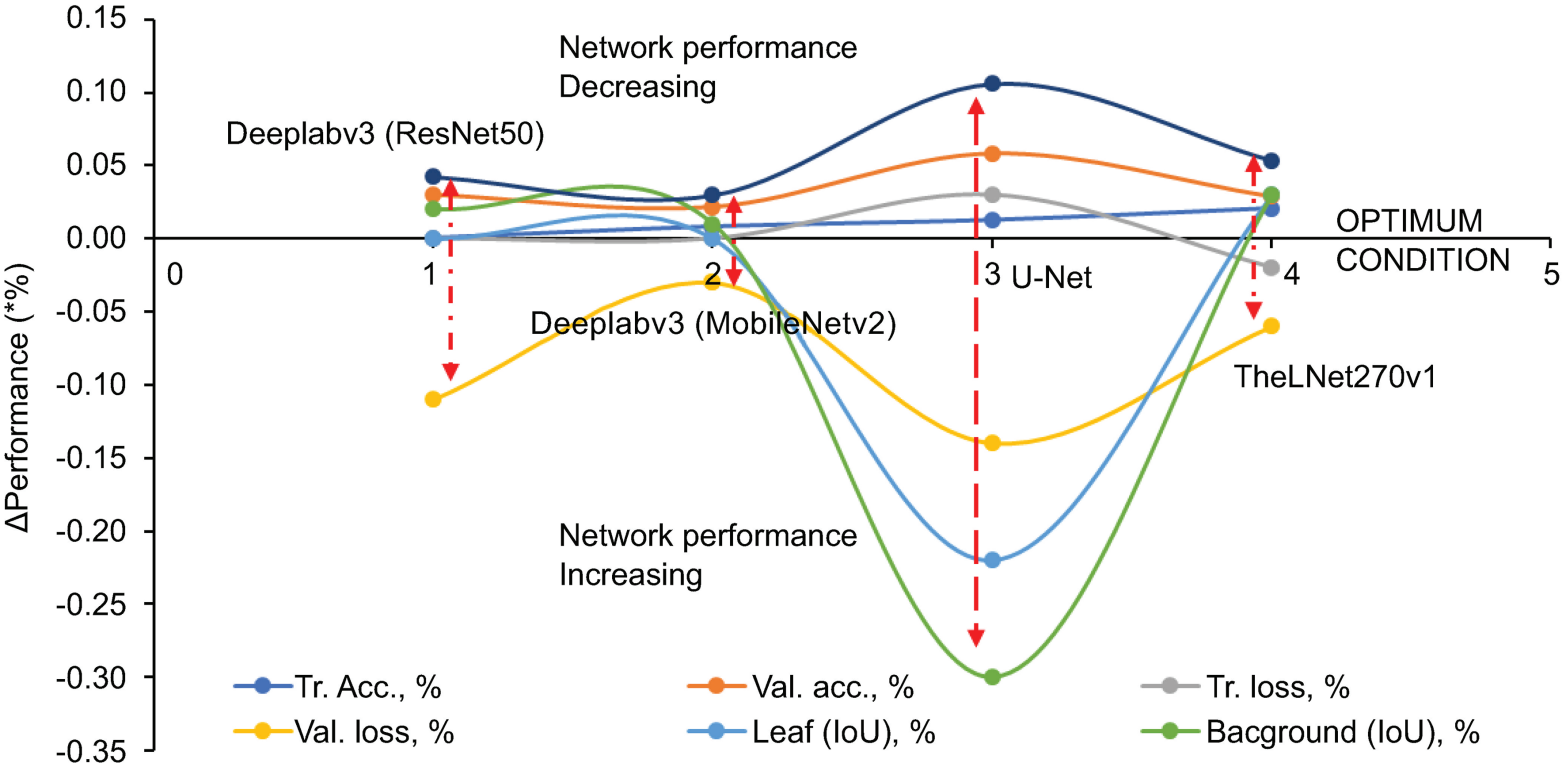
Δ Performance of the evaluation metrics.

Figure 12, Δ Performance (Eq. 1) demonstrated each evaluation metric’s positive and negative values with different image datasets. A negative value indicates an increase in the network performance, while a positive value is decreasing. The longer red arrow in the image indicates the volatile nature of the network due to the increase in the image dataset. From this, it is clear that Deeplabv3 (MobileNetv2) and TheLNet270v1 both show stable network performance despite increasing the number of images in the augmented dataset, as described in Table 6.

(1) $$\Delta \;Performance,\;\;\%=\left(\left(\begin{array}{l} evaluation\;metrics\;for\;\\ augmented\;data^{1}-\\ evaluation\;\;metrics\;\\ for\;\;augmented\;data^{2}/100 \end{array}\right)\times 100\right)$$

**Figure 13:**
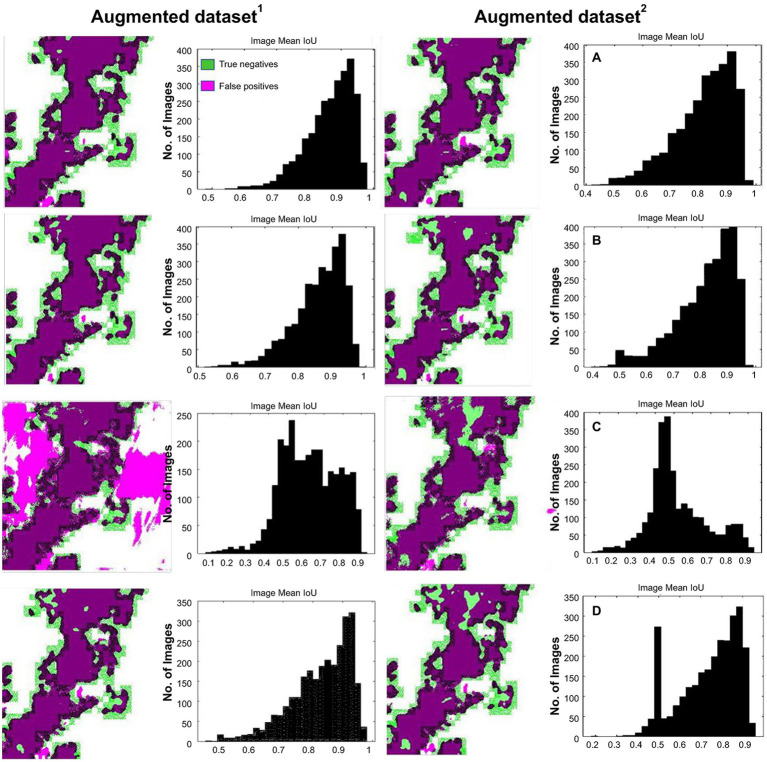
Test result vs. expected ground-truth (labeled) with intersection over union (IoU) histogram. (A) Deeplabv3plus (ResNet50). (B) Deeplabv3plus (MobileNetv2). (C) U-Net. (D) TheLNet270v1.

Test results vs. expected ground-truth (labeled) on the image-basis test dataset with IoU histogram are shown in Figures 13A–D [Deeplabv3plus (ResNet50), Deeplabv3plus (MobileNetv2), U-Net, and TheLNet270v1], and the mean IoU of each class, as described in Table 10. The mean IoU of the leaf and background classes is indicated by the top bar in the image histogram. Figure 13 and Table 10 show that the difference in mean IoU is clearly noticeable for the network trained with augmented dataset^1^ and augmented dataset^2^. No noticeable changes are occurring for networks trained with different types of data sets. However, U-Net demonstrated IoU improvement with an increasing number of image datasets. The results revealed that leaves that counted the maximum number of pixels had lower IoU than the background with the least number of pixels. Further increasing the number of images within the same pattern or adding high-resolution images can improve the network performance (Zhang et al., 2018).

**Table 10:** The mean IoU for comparative analysis.

Network name	Augmented data^1^	Augmented data^2^	Augmented data^1^	Augmented data^2^	Visualization
	Leaf		Background		
Deeplabv3plus (ResNet50)	0.74	0.74	0.87	0.85	Figure 13A
Deeplabv3plus (MobileNetv2)	0.72	0.72	0.86	0.85	Figure 13B
U-Net	0.44	0.66	0.52	0.82	Figure 13C
TheLNet270v1	0.73	0.7	0.87	0.84	Figure 13D

### Prediction Results

**Figure 14:**
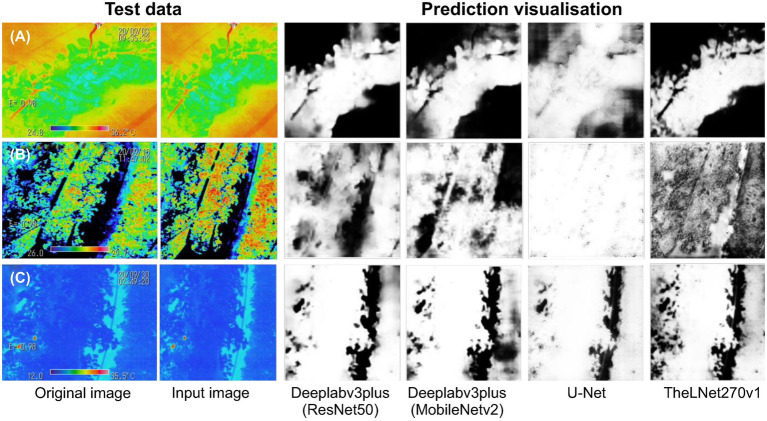
Prediction visualization of the single independent test image. (A) The early morning with a sunny condition. (B) The midday with a sunny–cloudy condition. (C) The midnight condition.

The prediction results of the independent image datasets are shown in Figure 14. Figure 14A represents the early morning with a sunny condition, Figure 14B represents the midday with a sunny–cloudy condition, and Figure 14C represents the midnight condition. These three sets of images were captured during September 2020 and were used to verify the network prediction efficiency. It is visualized that the TheLNet270v1 has better prediction ability compared with other networks.

### Network Performance Verification With the IPPN Plant Phenotyping Image Dataset

**Figure 15:**
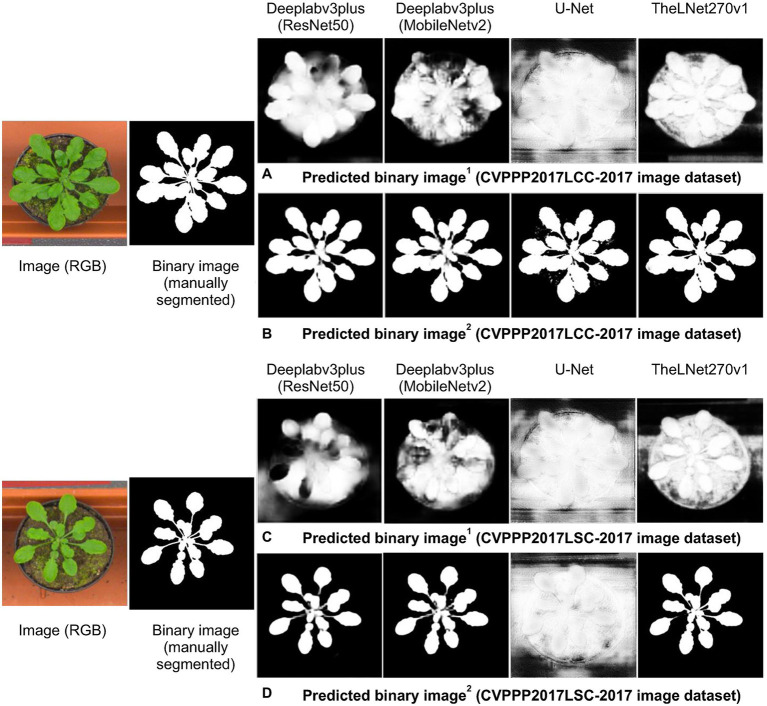
Verification of the prediction capability. (A) Comparison of manually segmented data with the predicted image from augmented 1 CVPPP2017LCC-2017 data. (B) Comparison of manually segmented data with the predicted image from augmented 2 CVPPP2017LCC-2017 data. (C) Comparison of manually segmented data with the predicted image from augmented 1 CVPPP2017LSC-2017 data. (D) Comparison of manually segmented data with the predicted image from augmented 2 CVPPP2017LSC-2017 data.

As we described earlier, thermal images of the greenhouse-grown tomato plants were used for training the TheLNet270v1. This network successfully classified leaf/canopy and its background with higher accuracy, as shown in Figures 11, 14. We further investigated TheLNet270v1 performance using the IPPN plant phenotyping image dataset (leaf segmentation challenge component of the CVPPP workshop: CVPPP2017LSC-2017 and CVPPP2017LCC-2017; Minervini et al., 2015), which included pot-cultivated *Arabidopsis thaliana*. First, we predicted the TheLNet270v1 output using image data from CVPPP2017LCC-2017 and CVPPP2017LSC-2017, as shown in Figures 15A,C. Subsequently, we trained the network with the CVPPP2017LSC-2017 image dataset (total images: 236, RGB) and then predicted again with the same image data from CVPPP2017LCC-2017 (Figures 15B,D). It is clearly visible that the TheLNet270v1 output, which is almost identical to the manually segmented binary image, is shown in Figure 15. Table 11 shows the TheLNet270v1 performance metrics.

**Table 11:** The performance metrics for image datasets.

Accuracy	Mean IoU, %	Weighted IoU, %	Mean BFScore, %
Train metrics	49.86	99.72	99.59
Validation metrics	49.84	99.68	99.61
Test metrics	49.91	99.81	99.81

IoU, intersection over union.

## Conclusion

This study introduced TheLNet270v1, a highly compact deep neural network (for mobile and non-mobile image classification) for classifying thermal images captured inside a greenhouse and demonstrating a higher classification accuracy. This paper also concludes a comparative analysis with other widely cited pre-trained networks for pixel-based classification, such as Deeplabv3plus (ResNet50), Deeplabv3plus (MobileNetv2), and U-Net, and found that TheLNet270v1 achieved a significantly better balance between accuracy and network efficiency. In our future work, we will apply the TheLNet270v1 network for on-site training, and output will be used for 24 h to monitor the relationships between plant growth and environmental conditions of the greenhouse. This network is suitable for the image with 240 × 240 pixels. However, to make it suitable for different pixel sizes, we consider modifying this network depending on the different image sizes in our future study.

## Data Availability Statement

The original contributions presented in the study are included in the article/supplementary material, further inquiries can be directed to the corresponding author.

## Author Contributions

MI performed the architecture development and analysis and wrote the first draft of the manuscript. MI, NK, and UL conducted the field experiment. KT and NK verified the experimental results. All authors contributed to manuscript revision and approved the submitted version.

### Conflict of Interest

The authors declare that the research was conducted in the absence of any commercial or financial relationships that could be construed as a potential conflict of interest.
